# Loneliness and well-being in finnish immigrants: A multimodal dataset from wearables and passive data collection

**DOI:** 10.1016/j.dib.2025.112331

**Published:** 2025-11-26

**Authors:** Yuning Wang, Jennifer Auxier, Amayag Mark, Parisa Farzanehkari, Anna Axelin, Iman Azimi, Amir M. Rahmani, Pasi Liljeberg

**Affiliations:** a University of Turku, Turku, Finland; b University of British Columbia, Vancouver, Canada; c University of California, Irvine, CA, USA

**Keywords:** Immigrants, Raw wearable physiological signals, Photoplethysmography (PPG) waveforms, Heart rate and heart rate variability, Self-reported real-life mental health data, Longitudinal sleep and physical activity, Ecological momentary assessments (EMAs)

## Abstract

This dataset was collected from first-generation immigrants between September 2022 and June 2023. Over a 28-day period, 39 participants aged 18 to 65, fluent in English and experiencing loneliness (UCLA Loneliness Scale score ≥ 28) contributed to the study. Data collection utilized Samsung Watch Active 2, Oura Ring, AWARE, and Centralive smartphone application. This dataset contains raw data from photoplethysmogram (PPG), inertial measurement unit (IMU) readings, air pressure, and processed data on heart rate, heart rate variability, sleep metrics (bedtime, stages, quality), physical activity (steps, active calories, activity types), and smartphone usage patterns (screen time, notifications, call and message logs). Participants also completed ecological momentary assessments (EMA) and weekly surveys, including instruments like the Beck Depression Inventory (BDI), Patient Health Questionnaire-9 (PHQ-9), Perceived Stress Scale, Sense of Coherence Scale, Social Connectedness Scale, Twente Engagement with E-Health Technologies questionnaire, and the UCLA Loneliness Scale. This dataset can be used to study the interplay between loneliness, mental well-being, and daily behaviors of immigrants in a real-world context.

Specifications Table

**Table:** 

Subject	Nursing, Psychology
Specific subject area	Mobile health monitoring
Type of data	Raw, Analyzed, Questionnaires, Table
Data collection	The data were collected from a wearable wristband smartwatch (Samsung Galaxy Active2), smart ring (Oura Ring), Questionnaire mobile application (Centralive app), mobile context logging application (AWARE). The data was collected from lonely immigrants living <5 years in Finland aged 18–65, whose smartphone is compatible with study devices and applications. Participants were ineligible if they are unable to speak/write English fluently.
Data source location	Institution: University of Turku City: Turku; Country: Finland
Data accessibility	Repository name:Dryad Data identification number: https://doi.org/10.5061/dryad.qz612jmrn;
Related research article	None

## 1 Value of the Data

• This dataset provides multimodal daily data on physiological, behavioral, and psychological measures collected over 28 days from first-generation immigrants in Finland, supporting studies on mental health and social adaptation.
• Researchers can use the data to examine how physiological patterns (e.g., sleep, activity, heart rate) and smartphone-based behaviors (e.g., call and message frequency, battery usage) interact with self-reported loneliness, stress, depression, connectedness, and feeling of isolation.
• The data enables modeling of temporal dynamics and identification of behavioral markers in emotional well-being or social connectedness.
• The dataset is suitable for developing and validating algorithmic approaches aimed at early detection or prevention of loneliness and stress in daily life.
• The data supports cross-cultural research on digital health, providing evidence for designing culturally sensitive interventions and public health strategies for immigrant populations.

## 2 Background

The dataset was created to address the lack of detailed data on loneliness and its related factors among first-generation immigrant populations. Based on the existing research, this dataset emphasizes the interconnection of physiological, psychological and social factors in shaping mental health outcomes. To capture this complexity, the dataset integrates wearable sensor data, passive smartphone data, EMA, and weekly surveys.

The dataset includes data from 39 participants aged 18–65 who engaged for 28 days data collection between September 2022 and June 2023. Every participant used a Samsung Watch and Oura Ring for monitoring cardiovascular health, sleep, and physical activity, alongside the AWARE app for passive smartphone data sensing, including call, message events, and screen activity. Daily EMAs and weekly surveys captured emotional states, including loneliness, depression and stress, via push notifications by Centralive — a custom-designed mobile app. Participants were recruited through purposive and snowball sampling. All participants met criteria including fluency in English, a UCLA Loneliness Scale score ≥28 (indicating loneliness), and ≤5 years of residence in Finland.

## 3 Data Description

The dataset provides a multimodal record of daily behaviors, physiological signals, and self-reported measures collected from first-generation immigrants in Finland over a 28-day period. Data are organized by participants in individual folders, each containing subfolders corresponding to the data sources (Aware, Oura, Watch, Survey). All data were de-identified prior to sharing, and the de-identification procedures are described in the Ethics Statement.

### 3.1 Population information

The general background information of the dataset is demonstrated in Table 1.

**Table 1:** Participants’ background information (*n* = 39).

Parameters	Values
Age, mean (SD)	31.5 (6.1)
Gender	
Female	26
Male	13
Months of residence in Finland, mean (SD)	17.7 (15.9)
Number of people around, mean (SD)	2.1 (1.2)
Finnish language skill	
None	8
Basic	26
Intermediate	5
Educational attainment	
High School Diploma	1
Some College	1
College Degree	11
Some Graduate School	2
Graduate Degree	24
Marital status	
Married	18
Single	14
Cohabitating	5
Divorced	1
Prefer not to say	1

### 3.2 Participants’ data

#### 3.2.1 Folder structure

At the root of the dataset directory tree, every participant has their own folder. These folders are named from “Participant_x”, where “x” represents every participant's unique number of identifiers. Every participant’s folder contains all the data collected from that participant, organized into subfolders based on different data sources used in the study. In each participant's folder, there are four subfolders corresponding to different data collection modalities: Aware, Oura, Watch, and Survey. Table 2 describes the folders in each participant’s folder. The following section provides a detailed description of the variables in each file and example visualizations.

**Table 2:** General folder and subfolder descriptions.

Subfolder name	Collected by	Description
Aware	AWARE framework	This folder contains passive data collected from the AWARE app, including various aspects of phone usage and behavioral data.
Oura	Oura ring v2	This folder holds data collected from the Oura Ring, covering metrics like sleep and physical activity.
Watch	Samsung Galaxy Active2 watch	This folder contains data from the Samsung Galaxy Active2 Watch, including raw signals captured by the accelerometer, gyroscope, gravity sensor, barometric pressure sensor, heart rate monitor, and photoplethysmogram (PPG) sensor.
Survey	Unite App	This folder contains various self-reported survey data files, organized by the type of questionnaire and the time of collection.

#### 3.2.2 Folder description and examples

##### 3.2.2.1 Aware

This folder contains passive data collected from the AWARE app, including various aspects of phone usage and behavioral data. The files include:

• battery: Logs on battery usage and charging events.
• calls: Data on incoming and outgoing calls.
• messages: Records of sent and received text messages.
• notifications: Information on received notifications.
• screen: Data on screen usage, such as screen-on and screen-off events.

**Fig. 1:**
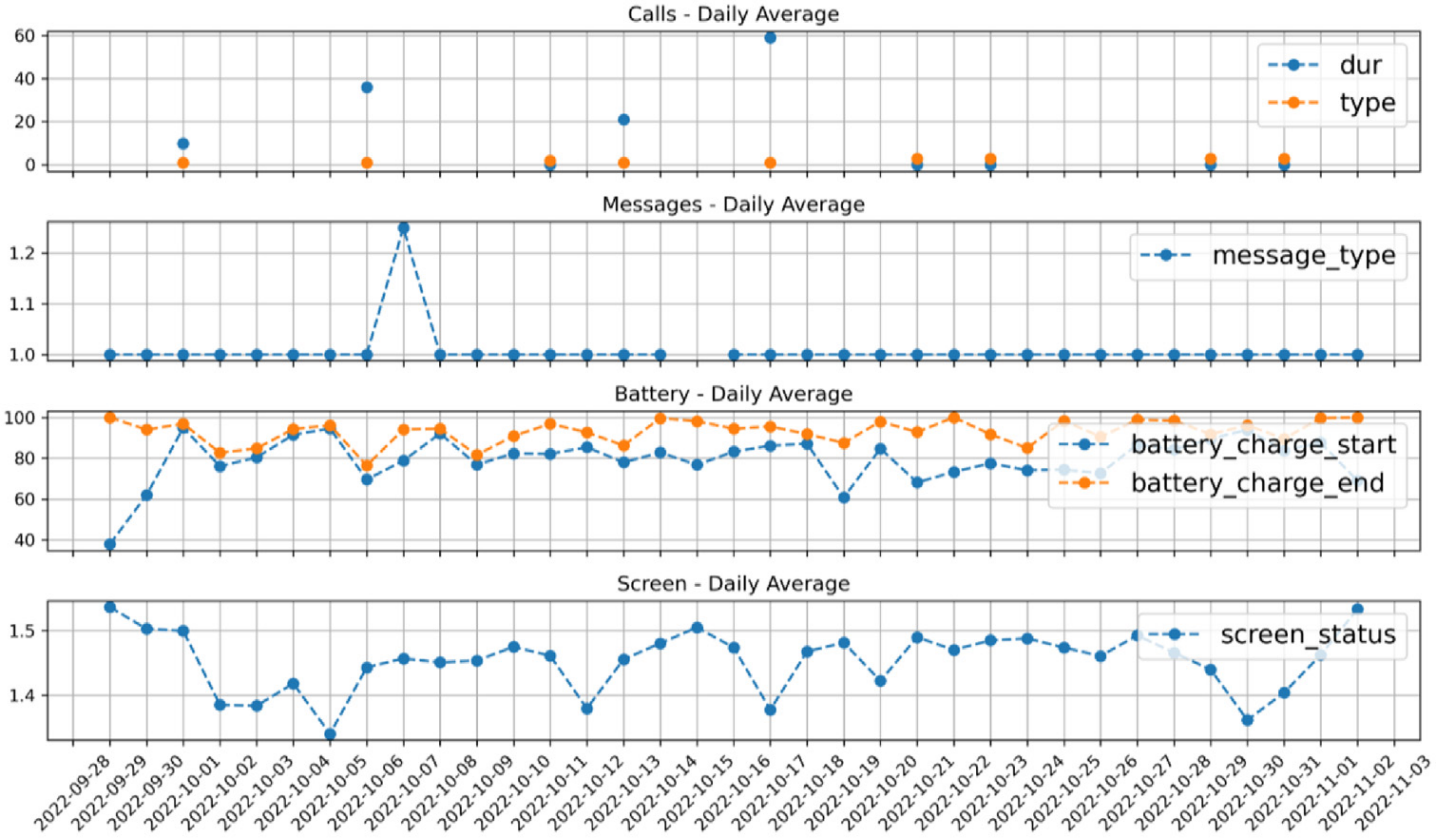
Daily average of selected features from aware data for one participant.

Descriptions of the variables within each file are provided in Table 3, Table 4, Table 5, Table 6, Table 7, and Fig. 1 presents the daily averages for one participant as an example visualization.

**Table 3:** Variables in battery.csv.

Column name	Description	Type of variable (Unit)
timestamp	13-digit timestamp	Timestamp (milliseconds)
participant	Participant ID	String
battery_charge_start	Percentage of the battery when starting to charge	Integer
battery_charge_end	Percentage of the battery when stopping the charge	Integer

**Table 4:** Variables in calls.csv.

Column name	Description	Type of variable (Unit)
timestamp	13-digit timestamp	Timestamp (milliseconds)
participant	Participant ID	String
dur	length of the call session	Integer
type	Android: 1 – incoming, 2 – outgoing, 3 – missed iOS: 1 – incoming, 2 – connected, 3 – dialing, 4 – disconnected	Integer

**Table 5:** Variables in messages.csv.

Column name	Description	Type of variable (Unit)
timestamp	13-digit timestamp	Timestamp (milliseconds)
participant	Participant ID	String
message_type	1 – received, 2 – sent	Integer

**Table 6:** Variables in notifications.csv.

Column name	Description	Type of variable (Unit)
timestamp	13-digit timestamp	Timestamp (milliseconds)
participant	Participant ID	String
package_category	Application’s category in Google Play Store	String

**Table 7:** Variable in screen.csv.

Column name	Description	Type of variable (Unit)
timestamp	13-digit timestamp	Timestamp (milliseconds)
participant	Participant ID	String
screen_status	0=off, 1=on, 2=locked, 3=unlocked	Integer

##### 3.2.2.2 Oura

This folder holds data collected from the Oura Ring, covering metrics like sleep and physical activity. The CSV files have been aligned by timestamp to ensure consistency across features. Variables containing ``sleep_5min'' in their names represent features sampled every five minutes while the participant was asleep, whereas those with ``activity_5min'' were sampled every five minutes throughout the day. Variables with “1min” were recorded every one minute throughout the day. All other features were recorded once per day.

**Fig. 2:**
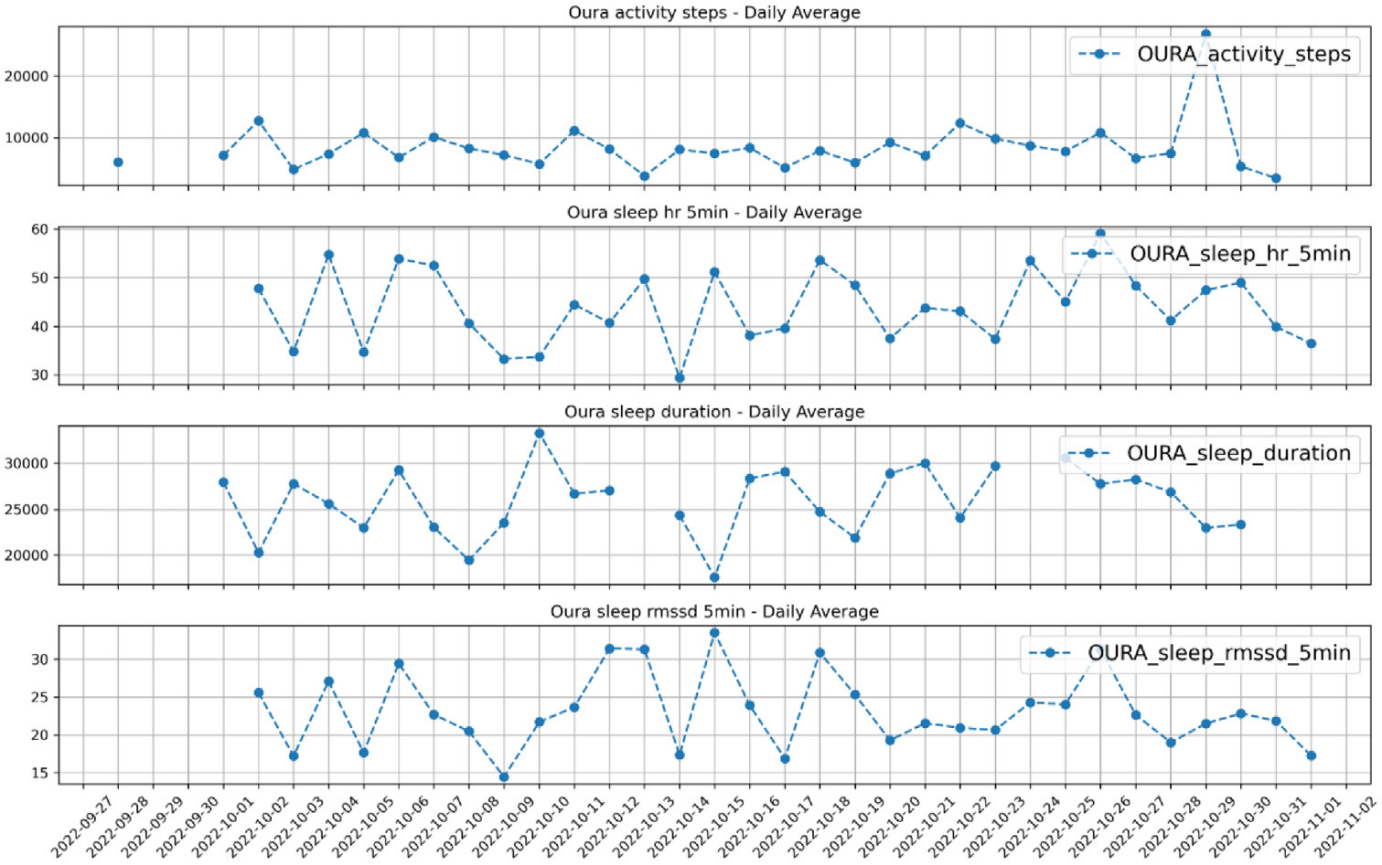
Daily average of selected features from oura data for one participant.

Descriptions for all Oura data fields used in this study follow the definitions provided in the Oura API Documentation [1]. For detailed definitions and data structures, please refer to the official documentation. A summary of the Oura variables included in this dataset is provided in Table 8, and Fig. 2 presents a visual example using four selected features (i.e., total steps, sleep average heart rate, sleep duration, and sleep average RMSSD)

**Table 8:** Variables in oura.csv.

Column name	Description	Type of variable (Unit)
timestamp	13-digit timestamp	Timestamp (milliseconds)
participant	Participant ID	String
OURA_activity_average_met	Average metabolic equivalent of task (MET), indicating the average activity intensity.	Float (calories)
OURA_activity_cal_active	Total calories burned during active periods.	Integer
OURA_activity_cal_total	Total calories burned	Integer
OURA_activity_class_5min	Activity classification data every 5 min: 0 non wear 1 rest 2 inactive 3 low activity 4 medium activity 5 high activity	Integer
OURA_activity_daily_movement	Daily total movement, usually measured in steps or distance traveled.	Integer
OURA_activity_high	Total time spent in high-intensity activity, measured in minutes.	Integer
OURA_activity_inactive	Total inactive time, measured in minutes.	Integer
OURA_activity_inactivity_alerts	Number of inactivity alerts triggered after prolonged inactivity.	Integer
OURA_activity_low	Total time spent in low-intensity activity, measured in minutes.	Integer
OURA_activity_medium	Total time spent in medium-intensity activity, measured in minutes.	Integer
OURA_activity_met_1min	Per-minute MET data.	Float array
OURA_activity_met_min_high	Total MET minutes for high-intensity activity.	Integer
OURA_activity_met_min_inactive	Total MET minutes for inactivity.	Integer
OURA_activity_met_min_low	Total MET minutes for low-intensity activity.	Integer
OURA_activity_met_min_medium	Total MET minutes for medium-intensity activity.	Integer
OURA_activity_non_wear	Total time when the device was not worn, measured in minutes.	Integer
OURA_activity_rest	Total time at rest, measured in minutes.	Integer
OURA_activity_rest_mode_state	Rest mode state, indicating whether the user was in rest mode.	Boolean
OURA_activity_score	Activity score, assessing overall daily activity level.	Integer
OURA_activity_score_meet_daily_targets	Score for meeting daily targets, evaluating if daily activity goals were achieved.	Integer
OURA_activity_score_move_every_hour	Score for moving every hour, evaluating if the user had activity each hour.	Integer
OURA_activity_score_recovery_time	Recovery time score, assessing user’s recovery status.	Integer
OURA_activity_score_stay_active	Stay active score, evaluating user’s consistent activity levels.	Integer
OURA_activity_score_training_frequency	Training frequency score, evaluating the user’s training frequency.	Integer
OURA_activity_score_training_volume	Training volume score, assessing the overall volume of user’s training.	Integer
OURA_activity_steps	Total steps, indicating the daily number of steps taken.	Integer
OURA_activity_target_calories	Target calories, user-defined goal for daily calorie burn.	Integer
OURA_activity_target_km	Target distance in kilometers, user-defined goal for daily walking or running distance.	Float
OURA_activity_target_miles	Target distance in miles, user-defined goal for daily walking or running distance.	Float
OURA_activity_to_target_km	Remaining distance to target in kilometers, indicating how much distance is left to reach the target.	Float
OURA_activity_to_target_miles	Remaining distance to target in miles, indicating how much distance is left to reach the target.	Float
OURA_activity_total	Total active time, measured in minutes.	Integer
OURA_ideal_bedtime_bedtime_window_end	End time of the ideal bedtime window.	Timestamp
OURA_ideal_bedtime_bedtime_window_start	Start time of the ideal bedtime window.	Timestamp
OURA_readiness_period_id	Unique identifier for the readiness period.	String
OURA_readiness_rest_mode_state	Readiness rest mode state, indicating whether the user was in rest mode.	Boolean
OURA_readiness_score	Readiness score, assessing the user’s overall recovery and readiness state.	Integer
OURA_readiness_score_activity_balance	Activity balance score, assessing balance between activity and rest.	Integer
OURA_readiness_score_hrv_balance	HRV balance score, assessing balance in heart rate variability.	Integer
OURA_readiness_score_previous_day	Previous day’s readiness score.	Integer
OURA_readiness_score_previous_night	Previous night’s readiness score.	Integer
OURA_readiness_score_recovery_index	Recovery index score, assessing user’s recovery speed.	Integer
OURA_readiness_score_resting_hr	Resting heart rate score, assessing resting heart rate health.	Integer
OURA_readiness_score_sleep_balance	Sleep balance score, assessing sleep quality and balance.	Integer
OURA_readiness_score_temperature	Temperature score, assessing stability and health of temperature.	Integer
OURA_sleep_average_breath_variation	Average breathing rate variation, assessing variation in breathing rate.	Float
OURA_sleep_awake	Total awake time during sleep, measured in minutes.	Integer
OURA_sleep_bedtime_end_delta	Difference between actual and planned wake-up time, measured in minutes.	Integer
OURA_sleep_bedtime_start_delta	Difference between actual and planned bedtime, measured in minutes.	Integer
OURA_sleep_breath_average	Average breathing rate, measured in breaths per minute.	Float
OURA_sleep_deep	Time spent in deep sleep, measured in minutes.	Integer
OURA_sleep_duration	Total sleep duration, measured in minutes.	Integer
OURA_sleep_efficiency	Sleep efficiency, ratio of total sleep time to time in bed.	Integer
OURA_sleep_got_up_count	Number of times user got up during sleep.	Integer
OURA_sleep_hr_5min	Heart rate data every 5 min during sleep.	Integer array
OURA_sleep_hr_average	Average heart rate during sleep.	Integer
OURA_sleep_hr_lowest	Lowest heart rate during sleep.	Integer
OURA_sleep_hypnogram_5min	Sleep stage data every 5 min.	String
OURA_sleep_is_longest	Indicates if the sleep period was the longest sleep session of the day.	Boolean
OURA_sleep_light	Time spent in light sleep, measured in minutes.	Integer
OURA_sleep_lowest_heart_rate_time_offset	Offset time to the lowest heart rate during sleep, measured in minutes.	Integer
OURA_sleep_midpoint_at_delta	Offset between actual and planned sleep midpoint, measured in minutes.	Integer
OURA_sleep_midpoint_time	Midpoint time of the sleep period.	Timestamp
OURA_sleep_onset_latency	Sleep latency, time taken to fall asleep, measured in minutes.	Integer
OURA_sleep_period_id	Unique identifier for the sleep period.	String
OURA_sleep_rem	REM sleep duration, measured in minutes.	Integer
OURA_sleep_restless	Restless time during sleep, indicating periods of disturbance.	Integer
OURA_sleep_rmssd	RMSSD value during sleep, assessing heart rate variability.	Float
OURA_sleep_rmssd_5min	RMSSD data every 5 min during sleep.	Float array
OURA_sleep_score	Sleep score, assessing overall sleep quality.	Integer
OURA_sleep_score_alignment	Sleep alignment score, assessing alignment of sleep time with ideal time.	Integer
OURA_sleep_score_deep	Deep sleep score, assessing quality of deep sleep.	Integer
OURA_sleep_score_disturbances	Disturbance score, assessing interruptions in sleep.	Integer
OURA_sleep_score_efficiency	Efficiency score, assessing effectiveness of sleep duration.	Integer
OURA_sleep_score_latency	Latency score, assessing time taken to fall asleep.	Integer
OURA_sleep_score_rem	REM sleep score, assessing quality of REM sleep.	Integer
OURA_sleep_score_total	Total sleep score, assessing overall sleep quality.	Integer
OURA_sleep_temperature_delta	Change in body temperature during sleep, measured in degrees Celsius.	Float
OURA_sleep_temperature_deviation	Temperature deviation during sleep, indicating deviation from normal body temperature.	Float
OURA_sleep_temperature_trend_deviation	Temperature trend deviation, indicating trend deviation over time.	Float
OURA_sleep_total	Total time spent in sleep, measured in minutes.	Integer
OURA_sleep_wake_up_count	Number of times user woke up during sleep.	Integer

##### 3.2.2.3 Watch

**Fig. 3:**
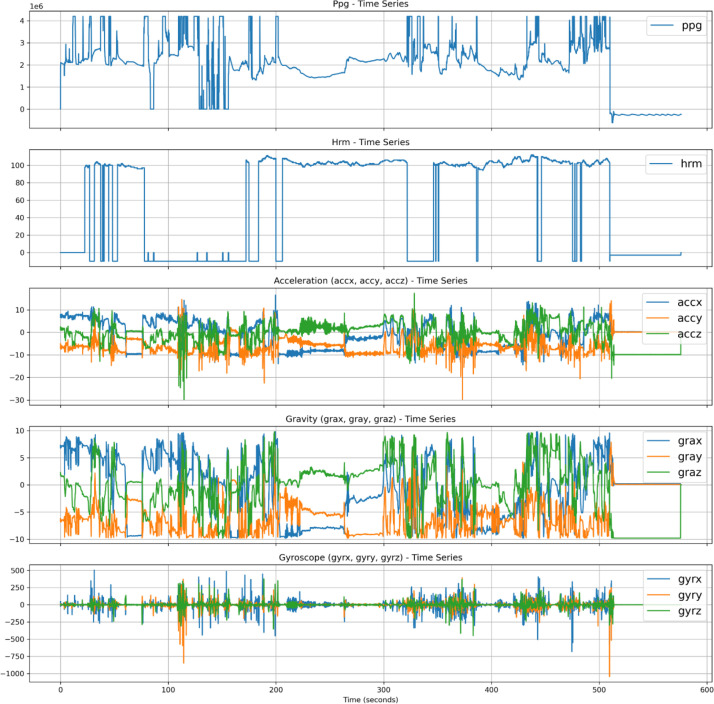
Selected features from watch data from one participant.

This folder contains data from the Samsung Galaxy Active2 Watch, including raw signals captured by the accelerometer, gyroscope, gravity sensor, barometric pressure sensor, heart rate monitor, and photoplethysmogram (PPG) sensor. The Watch folder includes two types of PPG recordings: “30sec_” files automatically collected during detected sleep events, and “data_” files representing scheduled 12-minute PPG recordings captured every two hours. A detailed description of the variables collected from each sensor is provided in Table 9. Fig. 3 presents visualizations of the raw signals from the sensors.

**Table 9:** Variables in watch data csv.

Column name	Description	Type
timestamp	13-digit timestamp	datetime
ppg	Photoplethysmography signal	Float
hrm	Heart rate measured in beats per minute (BPM).	Integer
accx	Acceleration in the x-axis	Float
accy	Acceleration in the y-axis	Float
accz	Acceleration in the z-axis	Float
grax	Gravity component in the x-axis	Float
gray	Gravity component in the y-axis	Float
graz	Gravity component in the z-axis	Float
gyrx	Gyroscope measurement in the x-axis	Float
gyry	Gyroscope measurement in the y-axis	Float
gyrz	Gyroscope measurement in the z-axis	Float
pressure	Atmospheric pressure	Float

##### 3.2.2.4 Survey

This folder contains various self-reported survey data files, organized by the type of questionnaire and the time of collection. The files include:

• **Beck Depression Inventory (BDI-II)**: A 21-item self-report scale assessing depressive symptoms (e.g., “I feel sad”). Each item is rated on a 4-point scale ranging from 0 (“not at all”) to 3 (“severe”), with total scores summed to represent depression severity [2]. The survey is administered at the beginning and end of the study.
• **Patient Health Questionnaire (PHQ-9)**: A 9-item measure of depressive symptoms (e.g., “Little interest or pleasure in doing things”), rated from 0 (“not at all”) to 3 (“nearly every day”) [3]. Weekly total scores were calculated as the sum of all items.
• **Perceived Stress Scale (PSS-4)**: A 4-item measure assessing perceived stress during the past week (e.g., “In the last week, how often have you felt that you were unable to control the important things in your life?”). Responses range from 0 (“never”) to 4 (“very often”), with total scores obtained by summing all items after reverse-coding the positive ones [4]. The PSS-4 was collected at the beginning, weekly, and end of the study.
• **Sense of Coherence Scale (SOC-13)**: A 13-item instrument measuring Meaningfulness (4 items), Comprehensibility (5 items), and Manageability (4 items) [5]. Each item is rated on a 7-point scale and the summed score reflects overall sense of coherence. Scores on SOC-13 were collected at the beginning and end of the study.
• **Social Connectedness Scale (SCS)**: An 8-item scale assessing interpersonal connectedness (e.g., “I feel disconnected from the world around me”), rated from 1 (“strongly disagree”) to 6 (“strongly agree”). Total scores were calculated as the sum of all items, with higher scores indicating lower social connectedness [6]. The SCS was collected at the beginning and the end of the study.
• **Twente Engagement with Ehealth Technologies (TWEETS)**: A 9-item measure of assessing the participant’s engagement with the eHealth technologies (e.g., “I think this MMML system could be a part of my daily routine”), rated from 0 (“strongly disagree”) to 4 (“strongly agree”) [7]. Summed scores were used to indicate engagement levels. The TWEETS were collected at the beginning, weekly, and end of the study.
• **UCLA Loneliness Scale (Version 3)**: A 20-item measure of loneliness and social isolation (e.g., “How often do you feel that you are “in tune” with the people around you?”), selecting from “always”, “sometimes”, “rare”, or “never” [8]. Total scores obtained by summing all items after reverse-coding the positive ones, with higher scores indicating greater loneliness. The UCLA scores were collected at the end of the study.
• **30-item Short Health Form Survey (SF-36):** a 36-item measure covering eight domains of health-related quality of life (i.e., Physical functioning, Role functioning/physical, Role functioning/emotional, Energy, Emotional well-being, Social functioning, Pain, and General health) [9]. The SF-36 was collected at the beginning and end of the study.
• **Ecological Momentary Assessments (EMA)**: Five single-item questions to capture momentary emotional and social states (i.e., loneliness, connectedness, isolatedness, positive feelings, and negative feelings). Each item was rated on a 0–10 scale. The EMAs are delivered 5 times per day via smartphone notifications. The EMA items were adapted from a prior EMA protocol that employed brief momentary single-item measures delivered multiple times per day [10].

The full wording and question index of all questionnaires are provided in Supplementary Material 1 for reference.

Descriptions of variables other than survey scale scores are provided in Table 10.

**Table 10:** Additional variables in survey files (Excluding raw scale scores).

Column name	Description	Type
timestamp	13-digit timestamp	datetime
participant	Participant ID	string
date	year-month-day	datetime
q#	The item-level response of the participant	string

#### 3.2.3 Additional notes

To further protect participant privacy while preserving the relative timing of events, all original timestamps (13 digits) were modified by applying a random offset for each individual. This procedure preserves the order and intervals of events, enabling analyses of temporal dynamics within participants (e.g., durations between events, clustering of activities).

## 4 Experimental Design, Materials and Methods

### 4.1 Design and set up

This study was designed to create a longitudinal dataset capturing physiological, behavioral, and psychological data from first-generation immigrants living in Finland. The dataset aims to support research on the relationship between mental health and daily lifestyle factors, providing a foundation for further detection algorithm development.

To achieve this, the study collected multimodal data over a 28-day period from every participant. Objective data were gathered from wearable devices, which recorded sleep patterns, physical activity, and cardiovascular health metrics and raw PPG signals. Passive smartphone data, such as screen usage, notifications, calls and messages, were also collected to capture digital behavior patterns.

Subjective data were collected through EMAs delivered via push notifications and weekly self-report surveys. These assessments measured daily emotional states—loneliness, stress, depression, and social connectedness. The sources and full references for each instrument are detailed in *Section 2.4 Surveys*. By integrating multiple data sources, this dataset allows researchers to explore the complex interactions between mental health and lifestyle behaviors under free-living conditions.

### 4.2 Data collection

To facilitate continuous data collection and remote monitoring, the Centralive was used [11]. Centralive is a digital health platform designed for continuous data collection, data storage, real-time monitoring, and remote management of participant engagement throughout the study. Data was collected using different applications, and wearable devices all centralized to the Centralive system. Then the collected data was transferred and stored in the Centralive’s cloud server. The Centralive’s dashboard was used to monitor the collected data to monitor participant’s engagement during data collection.

To collect the subjective daily EMAs and weekly surveys, the Centralive prompted the daily EMAs at 8 a.m., 2 pm., 5 pm., 8 pm., and 10 pm. to every participant. The daily EMA contains questions focusing on their current emotions including feelings of loneliness, social connectedness, and affect. The weekly EMA was open from 12 a.m. to 11:59 pm. and prompted participants every Sunday.

Samsung watch active 2 [12], equipped with Tizen open-source Operating System (TizenOS) was used to collect objective physiological signals. The device recorded photoplethysmography (PPG), accelerometer, and gyroscope data at a sampling rate of 20 Hz, while air pressure measurements were captured at 10 Hz. Data collection was scheduled at two-hour intervals, with each recording session lasting 12 min.

The Oura Ring was used to track participants' sleep and activity patterns throughout the study [13]. Data collected by the Oura Ring, including sleep, activity metrics, and cardiac metrics including heart rate and heart rate variability sensed during sleep. Centralive utilized Open Authentication to securely access and retrieve these data, making them available to researchers on a daily basis for further analysis.

The AWARE framework [14] was used to collect passive phone activity data. The AWARE app ran in the background on participants’ smartphones, continuously logging data without requiring active user input. The collected data included battery usage patterns, recording charging events and power consumption to monitor device usage trends. Call logs were also recorded, tracking incoming and outgoing calls with metadata such as timestamps and call duration, but without capturing conversation content. Similarly, message logs documented sent and received text messages, preserving metadata while ensuring privacy. Notifications data provided insights into participants’ digital engagement by logging received notifications, including app source and timestamps. Screen usage patterns were also recorded, capturing screen-on and screen-off events to estimate interaction frequency and duration.

### 4.3 Recruitment and enrollment

Participants were recruited between September 2022 and June 2023 through purposive and snowball sampling methods. Recruitment efforts included advertisements on selected social media platforms, language schools, and universities across Finland. Eligible participants were encouraged to recommend other first-generation immigrants who met the study criteria, expanding the recruitment network.

To be eligible for the study, participants had to meet the following criteria: (1) be between 18 and 65 years old, (2) be fluent in English, (3) have resided in Finland for no more than five years, and (4) experience loneliness, as indicated by a UCLA Loneliness Scale score of 28 or higher. A total of 42 participants initially enrolled, but three withdrew before completing the study. Therefore, data from the remaining 39 participants were included in the final dataset.

Upon expressing interest, potential participants were screened to confirm eligibility. Those who qualified were scheduled for an in-person enrollment session. During the session, participants were provided with detailed information about the study, and the research team reviewed the informed consent form before obtaining written consent. Participants then completed baseline psychological assessments, including surveys measuring loneliness, depression, stress, and social connectedness.

After enrollment, participants were guided through the study setup process. The research team assisted in configuring wearable devices, including the Samsung Watch Active 2 and the Oura Ring, and ensured that all necessary applications (AWARE, Oura, Galaxy Wearable) were installed on participants’ smartphones. Instructions were provided on how to use and maintain the devices throughout the study.

Participants were required to wear the devices daily and respond to EMAs and weekly surveys. The research team remotely monitored data collection through the Centralive platform to understand participant use and support and data integrity.

Participation in the study was voluntary. No financial or material compensation was offered to participants.

### 4.4 Exit

At the end of the 28-day study period, participants received a final set of surveys through Centralive. These surveys included the Beck Depression Inventory (BDI), the Patient Health Questionnaire-9 (PHQ-9), the Perceived Stress Scale, the Sense of Coherence Scale, the Social Connectedness Scale, the Twente Engagement with E-Health Technologies questionnaire, and the UCLA Loneliness Scale.

After completing these final assessments, participants were instructed to remove all study-related applications from their smartphones, including AWARE, Centralive, Oura, and Galaxy Wearable. They were also required to reset the Samsung Watch Active 2 and Oura Ring before returning the devices.

## Limitations

The dataset contains some data gaps due to technical and participant-related factors. Certain smartphone power-management settings limited continuous background recording, and wearable devices occasionally entered low-power modes under real-life usage, interrupting scheduled data collection. In addition, participants were informed they could remove the devices if these interfered with their daily activities, which in some cases resulted in inconsistent device usage.

This study did not provide any compensation for participation. Therefore, the dataset represents a sample of individuals who volunteered to contribute their time and effort over 28 days. This may introduce some degree of volunteer bias, as participants who were more motivated to take part in social activities.

The dataset is limited to first-generation immigrants in Finland who were fluent in English and had a UCLA Loneliness Scale score of 28 or higher. This selection criterion may limit generalizability to broader immigrant populations.

## Ethics Statement

This study was approved by the Ethics Committee for Human Sciences at the University of Turku (26/2022). Every participant provided a written informed consent before participating in the study. Before advertising on community webpages, Facebook, or through local universities, permission for each organization was sought, and recruitment was conducted according to their regulations. The sample of participants represented persons who were experiencing loneliness, this required that our research team was prepared to support and provide resources for individuals that showed signs or requested further support for their loneliness symptoms. Researchers had confidential and close contact with the participants, this enabled chances to observe for signs or signals that a participant would benefit from a referral to health services. No such need arose during the course of the study. In addition, information on resources available from community health support services was distributed to the participants.

To protect participant privacy and minimize the risk of re-identification, all data were de-identified prior to sharing. The following procedures were applied:

• All direct identifiers (e.g., names, contact information, device IDs) were removed.
• Each participant was assigned a pseudonymous identifier in the format Participant_x.
• Timestamp fields were shifted by a random offset to obscure exact dates while preserving within-participant temporal patterns.
• Application identifiers were generalized into broader categories (e.g., “social media app”).
• GPS location data were excluded.

## CRediT Author Statement

**Yuning Wang:** Methodology, Software, Formal analysis, Data curation, Writing – Original Draft; **Jennifer Auxier:** Investigation, Conceptualization, Validation, Formal analysis, Writing-Review Editing, Supervision; **Mark Amayag:** Investigation, Data curation, Validation; **Parisa Farzanehkari:** Investigation, Data curation, Validation; **Anna Axelin:** Conceptualization, Methodology, Investigation, Writing - Review & Editing, Supervision; **Iman Azimi:** Supervision; **Amir M. Rahmani:** Supervision; **Pasi Liljeberg:** Supervision.

## Data Availability

DryadLoneliness and well-being in Finnish immigrants: A multimodal dataset from wearables and passive data collection (Original data).

## Appendix Supplementary materials

Image, application 1
