# Supplementary material for: Loneliness and well-being in finnish immigrants: A multimodal dataset from wearables and passive data collection
Source: Data Brief. 2025 Nov 26;64:112331. doi: 10.1016/j.dib.2025.112331 (PMC12741416; doi:10.1016/j.dib.2025.112331)
Supplement: Supplementary file 1 [file mmc1.pdf]

Supplementary Materials

Ecological Momentary Assessment (EMA) Questions:

Lonely

1. How lonely do you feel right now? (rate on 0-10 sliding scale)

Social Connectedness/Social Isolation

1. How connected do you feel to others right now? (rate on a 1-10 sliding scale)

2. How isolated from others do you feel right now? (1-10)

Affect

1. How positive do you feel right now? Sliding scale (1-10)

2. How negative do you feel right now? Sliding scale (1-10)

UCLA Loneliness- 20 Item

1. How often do you feel that you are “in tune” with the people around you?

Always Sometimes Rarely Never

2. How often do you feel that you lack companionship?

Always Sometimes Rarely Never

3. How often do you feel that there is no one you can turn to?

Always Sometimes Rarely Never

4. How often do you feel alone?

Always Sometimes Rarely Never

5. How often do you feel part of a group of friends?

Always Sometimes Rarely Never

6. How often do you feel that you have a lot in common with the people around you?

Always Sometimes Rarely Never

7. How often do you feel that you are no longer close to anyone?

Always Sometimes Rarely Never

8. How often do you feel that your interests and ideas are not shared by those around you?

Always Sometimes Rarely Never

9. How often do you feel outgoing and friendly?

Always Sometimes Rarely Never

10. How often do you feel close to people?

Always Sometimes Rarely Never

11. How often do you feel left out?

Always Sometimes Rarely Never

12. How often do you feel that your relationships with others are not meaningful?

Always Sometimes Rarely Never

13. How often do you feel that no one really knows you well?

Always Sometimes Rarely Never

14. How often do you feel isolated from others?

Always Sometimes Rarely Never

15. How often do you feel you can find companionship when you want it?

Always Sometimes Rarely Never

16. How often do you feel that there are people who really understand you?

Always Sometimes Rarely Never

17. How often do you feel shy?

Always Sometimes Rarely Never

18. How often do you feel that people are around you but not with you?

Always Sometimes Rarely Never

19. How often do you feel that there are people you can talk to?

Always Sometimes Rarely Never

20. How often do you feel that three are people you can turn to?

Always Sometimes Rarely Never

Patient Health Questionnaire (PHQ-9)

(4-point scale, 0-3, not at all-nearly every day)

Over the last two weeks, how often have you been bothered by any of the following problems:

1. Little interest or pleasure in doing things
2. Feeling down, depressed, or hopeless
3. Trouble falling asleep, staying asleep, or sleeping too much
4. Feeling tired or having little energy
5. Poor appetite or overeating
6. Feeling bad about yourself - or that you are a failure or have let yourself or your family down
7. Trouble concentrating on things, such as reading the newspaper or watching television
8. Moving or speaking so slowly that other people could have noticed? Or the opposite - being so fidgety or restless that you have been moving around a lot more than usual
9. Thoughts that you would be better off dead or hurting yourself in some way

Beck's Depression Inventory (BDI-II)

1.

0 I do not feel sad

1 I feel sad

2 I am sad all the time and I can’t snap out of it

3 I am so sad and unhappy that I can’t stand it

2.

0 I am not particularly discouraged about the future

1 I feel discouraged about the future

2 I feel I have nothing to look forward to

3 I feel the future is hopeless and that things cannot improve

3.

0 I do not feel like a failure

1 I feel I have failed more than the average person

2 As I look back on my life, all I can see is a lot of failures

3 I feel I am a complete failure as a person

4.

0 I get as much satisfaction out of things as I used to

1 I don’t enjoy things the way I used to

2 I don’t get real satisfaction out of anything anymore

3 I am dissatisfied or bored with everything

5.

0 I don’t feel particularly guilty

1 I feel guilty a good part of the time

2 I feel quite guilty most of the time

3 I feel guilty all of the time

6.

0 I don’t feel I am being punished

1 I feel I may be punished

2 I expect to be punished

3 I feel I am being punished

7.

0 I don’t feel disappointed in myself

1 I am disappointed in myself

2 I am disgusted with myself

3 I hate myself

8.

0 I don’t feel I am any worse than anybody else

1 I am critical of myself for my weaknesses or mistakes

2 I blame myself all the time for my faults

3 I blame myself for everything bad that happens

9.

0 I don’t have any thoughts of killing myself

1 I have thoughts of killing myself, but I would not carry them out

2 I would like to kill myself

3 I would kill myself if I had the chance

10.

0 I don’t cry any more than usual

1 I cry more now than I used to

2 I cry all the time now

3 I used to be able to cry, but now I can’t cry even though I want to

11.

0 I am no more irritated by things than I ever was

1 I am slightly more irritated now than usual

2 I am quite annoyed or irritated a good deal of the time

3 I feel irritated all the time

12.

0 I have not lost interest in other people

1 I am less interested in other people than I used to be

2 I have lost most of my interest in other people

3 I have lost all of my interest in other people

13.

0 I make decisions about as well as I ever could

1 I put off making decisions more than I used to

2 I have greater difficulty in making decisions more than I used to

3 I can't make decisions at all anymore

14.

0 I don't feel that I look any worse than I used to

1 I am worried that I am looking old or unattractive

2 I feel there are permanent changes in my appearance that make me look unattractive

3 I believe that I look ugly

15.

0 I can work about as well as before

1 It takes an extra effort to get started at doing something

2 I have to push myself very hard to do anything

3 I can't do any work at all

16.

0 I can sleep as well as usual

1 I don't sleep as well as I used to

2 I wake up 1-2 hours earlier than usual and find it hard to get back to sleep

3 I wake up several hours earlier than I used to and cannot get back to sleep

17.

0 I don't get more tired than usual

1 I get tired more easily than I used to

2 I get tired from doing almost anything

3 I am too tired to do anything

18.

0 My appetite is no worse than usual

1 My appetite is not as good as it used to be

2 My appetite is much worse now

3 I have no appetite at all anymore

19.

0 I haven’t lost much weight, if any, lately

1 I have lost more than 2 kilograms

2 I have lost more than 5 kilograms

3 I have lost more than 7 kilograms

20.

0 I am no more worried about my health than usual

1 I am worried about physical problems like aches, pains, upset stomach, or constipation

2 I am very worried about physical problems and it’s hard to think of much else

3 I am so worried about my physical problems that I cannot think of anything else

21.

0 I have not noticed any recent change in my interest in sex

1 I am less interested in sex than I used to be

2 I have almost no interest in sex

3 I have lost interest in sex completely

Perceived Stress Scale (PSS-4)

(5-point scale; 0-4, never-very often)

In the last week, how often have you felt:

1. That you were unable to control the important things in your life?

2. Difficulties were piling up so high that you could not overcome them?

3. Confident about your ability to handle your personal problems?

4. How often have you felt that things were going your way?

Sense of Coherence Scale (SOC-13)

1. Do have the feeling that you don't really care about what goes on around you?

1	2	3	4	5	6	7
Very seldom or never						Very often

2. Has it happened in the past that you were surprised by the behavior of people whom you thought you knew well?

1	2	3	4	5	6	7
Never happened						Always happened

3. Has it happened that people whom you counted on disappointed you?

1	2	3	4	5	6	7
Never happened						Always happened

4. Until now your life has had:

1	2	3	4	5	6	7
No clear goals or purpose at all						Ver clear goals and purpose

5. Do you have the feeling that you're being treated unfairly?

1	2	3	4	5	6	7
Very often						Very seldom or never

6. Do you have the feeling that you are in an unfamiliar situation and don't know what to do?

1	2	3	4	5	6	7
Very often						Very seldom or never

7. Doing the things you do every day is:

1	2	3	4	5	6	7
A source of deep pleasure and satisfaction						A source of pain and boredom

8. Do you have very mixed-up feelings and ideas?

1	2	3	4	5	6	7
Very often						Very seldom or never

9. Does it happen that you have feelings inside you would rather not feel?

1	2	3	4	5	6	7
Very often						Very seldom or never

10. Many people - even those with a strong character - sometimes feel like sad sacks (losers) in certain situations. How often have you felt this way in the past?

1	2	3	4	5	6	7
Never						Very often

11. When something happened, have you generally found that:

1	2	3	4	5	6	7
You overestimated or underestimated its importance						You saw things in the right proportion

12. How often do you have the feeling that there's little meaning in the things you do in your daily life?

1	2	3	4	5	6	7
Very often						Very seldom or never

13. How often do you have feelings that you're not sure you can keep under control?

1	2	3	4	5	6	7
Very often						Very seldom or never

Social Connectedness Scale

1. I feel disconnected from the world around me

1 2 3 4 5 6

Agree Disagree

2. Even around people I know, I don't feel that I really belong

1 2 3 4 5 6

Agree Disagree

3. I feel so distant for people

1 2 3 4 5 6

Agree Disagree

4. I have no sense of togetherness with my peers

1 2 3 4 5 6

Agree Disagree

5. I don't feel related to anyone

1 2 3 4 5 6

Agree Disagree

6. I catch myself losing all sense of connectedness with society

1 2 3 4 5 6

Agree Disagree

7. Even among my friends, there is no sense of brother/sisterhood

1 2 3 4 5 6

Agree Disagree

8. I don't feel I participate with anyone or any group

1 2 3 4 5 6

Agree Disagree

Twente Engagement with Ehealth Technologies (TWEETS)

5-point Likert scale (strongly disagree=0, disagree=1, neutral=2, agree=3, strongly agree=4)

1. This MMML system is part of my daily routine

2. This MMML system is easy to use

3. I am able to use this MMML system as often as needed (to achieve my goals of becoming less lonely)

4. This MMML system makes it easier for me to work on becoming less lonely

5. This MMML system motivates me to reach my goal of becoming less lonely

6. This MMML system helps me to get more insight into my behavior related to becoming less lonely

7. I enjoy using the MMML system

8. I enjoy seeing the progress I make with the MMML system

9. This MMML system fits me as a person

30-item Short Health Form Survey (SF-36)

Please answer the 36 questions of the Health Survey completely, honestly, and without interruptions.

GENERAL HEALTH:

In general, would you say your health is:

__Excellent __Very Good __Good__Fair __Poor

Compared to one year ago, how would you rate your health in general now?

__Much better now than one year ago

__Somewhat better now than one year ago

__About the same

__Somewhat worse now than one year ago

__Much worse than one year ago

LIMITATIONS OF ACTIVITIES:

The following items are about activities you might do during a typical day. Does your health now limit you in these activities? If so, how much?

Vigorous activities, such as running, lifting heavy objects, participating in strenuous sports.

__Yes, Limited a lot __Yes, Limited a Little __No, Not Limited at all

Moderate activities, such as moving a table, pushing a vacuum cleaner, bowling, or playing golf

__Yes, Limited a lot __Yes, Limited a Little __No, Not Limited at all

Lifting or carrying groceries

__Yes, Limited a lot __Yes, Limited a Little __No, Not Limited at all

Climbing several flights of stairs

__Yes, Limited a lot __Yes, Limited a Little __No, Not Limited at all

Climbing one flight of stairs

__Yes, Limited a lot __Yes, Limited a Little __No, Not Limited at all

Bending, kneeling, or stooping

__Yes, Limited a lot __Yes, Limited a Little __No, Not Limited at all

Walking more than a mile

__Yes, Limited a lot __Yes, Limited a Little __No, Not Limited at all

Walking several blocks

__Yes, Limited a lot __Yes, Limited a Little __No, Not Limited at all

Walking one block

__Yes, Limited a lot __Yes, Limited a Little __No, Not Limited at all

Bathing or dressing yourself

__Yes, Limited a lot __Yes, Limited a Little __No, Not Limited at all

PHYSICAL HEALTH PROBLEMS:

During the past 4 weeks, have you had any of the following problems with your work or other regular daily activities as a result of your physical health?

Cut down the amount of time you spent on work or other activities

__Yes __No

Accomplished less than you would like

__Yes __No

Were limited in the kind of work or other activities

__Yes __No

Had difficulty performing the work or other activities (for example, it took extra effort)

__Yes __No

EMOTIONAL HEALTH PROBLEMS:

During the past 4 weeks, have you had any of the following problems with your work or other regular daily activities as a result of any emotional problems (such as feeling depressed or anxious)?

Cut down the amount of time you spent on work or other activities

__Yes __No

Accomplished less than you would like

__Yes __No

Didn't do work or other activities as carefully as usual

__Yes __No

SOCIAL ACTIVITIES:

Emotional problems interfered with your normal social activities with family, friends, neighbors, or groups?

__Not at all __Slightly Moderately __Severe __Very Severe

PAIN:

How much bodily pain have you had during the past 4 weeks?

__Not at all __Slightly Moderately __Severe __Very Severe

During the past 4 weeks, how much did pain interfere with your normal work (including both work outside the home and housework)?

__Not at all __A little bit __Moderately __Quite a bit __Extremely

ENERGY AND EMOTIONS:

These questions are about how you feel and how things have been with you during the last 4 weeks. For each question, please give the answer that comes closest to the way you have been feeling.

Did you feel full of pep?

__All of the time

__Most of the time

__A good Bit of the Time

__Some of the time

__A little bit of the time

__None of the Time

Have you been a very nervous person?

__All of the time

__Most of the time

__A good Bit of the Time

__Some of the time

__A little bit of the time

__None of the Time

Have you felt so down in the dumps that nothing could cheer you up?

__All of the time

__Most of the time

__A good Bit of the Time

__Some of the time

__A little bit of the time

__None of the Time

Have you felt calm and peaceful?

__All of the time

__Most of the time

__A good Bit of the Time

__Some of the time

__A little bit of the time

__None of the Time

Did you have a lot of energy?

__All of the time

__Most of the time

__A good Bit of the Time

__Some of the time

__A little bit of the time

__None of the Time

Have you felt downhearted and blue?

__All of the time

__Most of the time

__A good Bit of the Time

__Some of the time

__A little bit of the time

__None of the Time

Did you feel worn out?

__All of the time

__Most of the time

__A good Bit of the Time

__Some of the time

__A little bit of the time

__None of the Time

Have you been a happy person?

__All of the time

__Most of the time

__A good Bit of the Time

__Some of the time

__A little bit of the time

__None of the Time

Did you feel tired?

__All of the time

__Most of the time

__A good Bit of the Time

__Some of the time

__A little bit of the time

__None of the Time

SOCIAL ACTIVITIES:

During the past 4 weeks, how much of the time has your physical health or emotional problems interfered with your social activities (like visiting with friends, relatives, etc.)?

__All of the time

__Most of the time

__A good Bit of the Time

__Some of the time

__A little bit of the time

__None of the Time

GENERAL HEALTH:

How true or false is each of the following statements for you?

I seem to get sick a little easier than other people

__Definitely true __Mostly true __Don't know __Mostly false __Definitely false

I am as healthy as anybody I know

__Definitely true __Mostly true __Don't know __Mostly false __Definitely false

I expect my health to get worse

__Definitely true __Mostly true __Don't know __Mostly false __Definitely false

My health is excellent

__Definitely true __Mostly true __Don't know __Mostly false __Definitely false
